# The influence of internet use frequency, family atmosphere, and academic performance on adolescent depression: Based on the chain mediating effect of self-adjustment and campus deviant behavior

**DOI:** 10.3389/fpsyg.2022.992053

**Published:** 2022-12-05

**Authors:** Mao-min Jiang, De-wen Wang, Zheng-yu Wu, Kai Gao, Pei-pei Guo, Yang Kong

**Affiliations:** ^1^School of Public Affairs, Xiamen University, Xiamen, China; ^2^School of Management, Shanghai University of Engineering Sciences, Shanghai, China; ^3^School of Public Health and Management, Binzhou Medical University, Yantai, China

**Keywords:** family atmosphere, academic performance, self-control, problem behavior, depressive symptoms, chain mediation, Internet use frequency

## Abstract

**Introduction:**

Depression has become a prominent psychological problem among young people. The purpose of this study was to investigate the potential relationship between the frequency of Internet use, family atmosphere, academic performance, self-adjustment, campus deviant behavior, and depressive symptoms among adolescents.

**Methods:**

Based on the survey data of the fifth wave (2017~2018) and the sixth wave (2019~2020) of the China Family Panel Studies (CFPS), this study used LISREL8.8 software to analyze 1,577 10~15 data on adolescents.

**Results:**

In this study, the mean score of self-adjustment was 42.40 (SD = 6.79), the mean score of campus deviant behavior was 12.59 (SD = 4.00), the mean score of depressive symptoms in 2018 was 11.88 (SD = 3.04), and the mean score of depressive symptoms in 2020 was 7.64 (SD = 2.20). Secondly, the frequency of Internet use had no direct effect on the depressive symptoms of adolescents, family atmosphere was negatively correlated with depressive symptoms (*p* < 0.005), and academic performance was positively correlated with depressive symptoms (*p* < 0.005). Depressive symptoms in 2020 had a direct effect (*β* = 0.37, *p* < 0.001), and also had a negative effect on depressive symptoms in 2020, with a total effect of-0.07 (*p* < 0.001); self-adjustment had no direct effect on depressive symptoms in adolescents in 2018, However, the total effect was −0.14 (*p* < 0.001), which had a significant positive effect on 2020 depressive symptoms, and the total effect was 0.18 (*p* < 0.001), and self-adjustment had a significant negative effect on adolescent campus deviant behavior (*β* = −0.38, *p* < 0.001); in addition, the frequency of Internet use, family atmosphere, and academic performance all had indirect effects on adolescents’ 2020 depressive symptoms, with total effects of −0.60, 0.01, and 0.02 (*p* < 0.001), respectively. This study also found depressive symptoms in adolescents have a certain persistence in time.

**Discussion:**

Based on this study, it is necessary to pay more attention to the depression of adolescents, strengthen the training of self-adjustment, improve the anti-frustration ability and psychological resilience, and reduce the campus deviant behavior of adolescents. It is recommended to try to start from emotional self-adjustment to promote the personality health of adolescents.

## Background

Depression has become one of the most important psychological problems in today’s world (Pooja and Mihir, 2021). It can cause people to feel depressed, and reduce their activity and language movements (Smith, 2014). Studies have shown that depression symptoms are caused by emotional disorder, which leads to physical and mental discomfort in individuals. Specific manifestations include sadness, despair, other emotions, and even suicidal tendencies (Cheung and Dewa, 2007). According to the results of the World Health Organization survey, at present, about 4.4% of the world’s population suffers from depression, and more than 300 million people suffer from depression [World Health Organization (WHO), 2017]. Some studies have also found that in recent years, depression has gradually become younger, and there is an increasing number that young people suffering from depression, among which the depression of adolescents is particularly prominent (ServanMori et al., 2020) Adolescents suffering from depression not only affect their physical and mental development but also destroy family harmony, which is not conducive to social harmony and stability. Therefore, it is particularly important to explore the mechanism, possible influencing factors, and development path of adolescent depressive symptoms.

The Internet is a double-edged sword. Long-term addiction to the Internet will not only cause personal psychological obstacles, but also affect their normal living conditions (Davis, 2001). A large number of studies show that frequency of Internet use is closely related to depression, and the frequency of Internet use rate of depressed people is significantly higher than that of normal people (Li et al., 2019). Studies have found that Depressed patients with pessimism and anxiety generally have a low willingness to participate in social activities, while the social mode of virtual space can effectively avoid the friction and harm when people face to face with each other. More depressed people are more inclined to socialize online. Based on the substitution of online socialization, Depressed individuals use the Internet more often (Kraut et al., 1998; Scott et al., 2009); meanwhile, Depressed people will regulate their emotions through online media such as online shopping, watching videos, and playing games (Kim et al., 2017). Secondly, some studies have found that frequency of Internet use is an important factor affecting depression. Long-term Internet use will reduce the communication between relatives and friends, reduce personal social adaptation, and fail to get enough social support. Leading to depression (Liang et al., 2016). In addition, there is also a scholar who believes that there is no direct correlation between depression and frequency of Internet use. Although depressed patients can compensate for individual interpersonal communication by using the Internet, it also reduces offline interaction (Anderson, 2001; Yao and Zhong, 2014). And they even think that frequency of Internet use can alleviate the depression of individuals. To some extent, the use of the Internet makes up for the social deficiency of depressed people (Przepiorka et al., 2019). Although there are many research on frequency of Internet use and depression, the relationship between them is still worth further exploring.

As the most primitive environment for an individual to grow up in, the family is generally a system unit formed by the interaction between parents and children. In this simple but complex set unit, the influence of the family atmosphere on individuals is self-evident (LeCloux et al., 2016). Relevant research shows that adolescent depression is closely related to the family atmosphere (Ribeiro et al., 2016). The family model theory holds that the better the atmosphere and environment of the family system, the better the family function, the more flexible the psychological quality and behavior of family members, and a bad family atmosphere will also lead to the risk of depression (Robert and Robert, 2000). The hopeless theory of depression also reflects that the family atmosphere has a negative effect on individual depression (Abramson et al., 1989). Adolescents have strong emotional dependence and emotional impulsiveness, and they are a high incidence of depression, and a good family atmosphere is extremely important for adolescents’ emotional guidance and the cultivation of their ability to resist setbacks (Alison et al., 1998). Harmonious family relationships can promote adolescents’ sense of social belonging, and positive parent–child interaction can also help adolescents enhance their psychological resilience (Guo, 2018). However, there is a complex endogenous relationship between adolescents’ psychological state, emotional color, and depression. Therefore, it is of great significance to explore the influence path of the family atmosphere on depression.

Based on China’s education system, social culture, expectations of parents and teachers, and peer pressure, adolescents are more likely to suffer from depression due to their academic performance (Zhang et al., 2015); many studies have shown that there is a direct correlation between academic performance and depressive symptoms (Katherine et al., 2012; Verboom et al., 2013). Some studies believe that low academic performance is more likely to produce negative emotions (Pomerantz et al., 2002). In primary and secondary schools, adolescents’ lack of academic performance, learning attitude, learning style, etc. leads to negative events, and negative feedback from parents, teachers, and classmates, resulting in self-denial psychology, which makes them more prone to depression. On the other hand, Some scholars also believe that adolescents with good academic performance are at greater risk of depression. The essence of adolescent depression is the lack of self-worth, and losing self-worth is easy to lose self-confidence, thus falling into depression. Adolescents with good academic performance, parents, teachers, and self-expectations are relatively high, and academic achievement becomes the main source of their sense of value. Therefore, Compared to those with poor academic performance, 90% of them are under greater learning pressure, which makes them more vulnerable to the gap, thus increasing the risk of depression (Çelik, 2019). Therefore, the relationship between academic performance and depression needs to be further verified.

The campus deviant behavior of adolescents refers to the violation of the rules and regulations of the campus and the general behavior. The external reasons for the deviant behavior mainly include factors such as family environment disorder, bad social environment, and incorrect value guidance. The internal reasons are mainly Individual physical development and psychological development is not matched, and social adaptability and frustration tolerance are insufficient (Markova and Nikitskaya, 2014). Campus deviant behaviors can be divided into internalized deviant behaviors and externalized deviant behaviors. Internalized deviant behaviors generally refer to emotions such as anxiety, sadness, and withdrawal caused by lack of attention. Externalized deviant behaviors mainly refer to behavioral problems, such as attack, rage, etc. (Myers et al., 1994). Some studies have shown that long-term deviant behaviors in schools can easily lead to extreme behaviors among adolescents, resulting in immeasurable losses (Benda and Corwyn, 1998). Some studies have found that adolescents with high Internet use are more likely to develop school-biased behaviors (Lin et al., 2020). The “people in the context” perspective theory found that parent–child relationship is an important factor in reducing adolescents’ school-biased behaviors (Pavkov et al., 2010; Liu, 2016). The social learning theory proposes that teenagers are affected by the social environment in the learning process. The greater the learning pressure, the worse the academic performance, and the more easily they are ignored by teachers, the more likely they are to have a deviant behavior, hoping to win the attention and attention of teachers and students (Ma et al., 2018; Çelik, 2019). However, the comprehensive theoretical model of deviant behavior believes that adolescent deviant behavior is persistent and can significantly affect adolescent violence behavior and even depressive suicide behavior in adulthood. This not only affects the individual health of young people, but also is not conducive to the harmonious and stable development of society (Jiang et al., 2022). Therefore, whether there is a mediating relationship among adolescents’ Internet use frequency, family atmosphere, academic performance, and depressive symptoms needs further verification.

Self-adjustment generally refers to self-reinforcing, that is, the process of reinforcing and maintaining one’s behavior with rewards that can be controlled when people achieve their own standards (Inzlicht et al., 2014). It has been found that the higher the self-adjustment ability, the higher the adjustment ability of an individual’s stress ability and emotional response, and the negative behavior can be reduced when negative feedback is received (Glenn, 2000; Finning et al., 2017). At present, more studies have introduced self-adjustment into the field of psychology to improve addiction or aggressive behavior by training self-adjustment ability (Remster, 2014). Related studies have found that the higher the self-adjustment level of adolescents, the less depressed they are (Jun and Choi, 2013; Yang et al., 2017). At the same time, self-adjustment can also reduce family conflicts and poor academic performance to a certain extent (Li, 2004; Reisig and Pratt, 2011). In addition, relevant studies also show that self-adjustment is an important factor affecting the campus deviant behavior of adolescents, and it plays an important mediating role in the influence of other factors on the campus deviant behavior of adolescents, such as parent–child relationship and social support (Cho et al., 2017). In view of this, this study will also explore the mediating role of self-adjustment.

**Figure 1:**
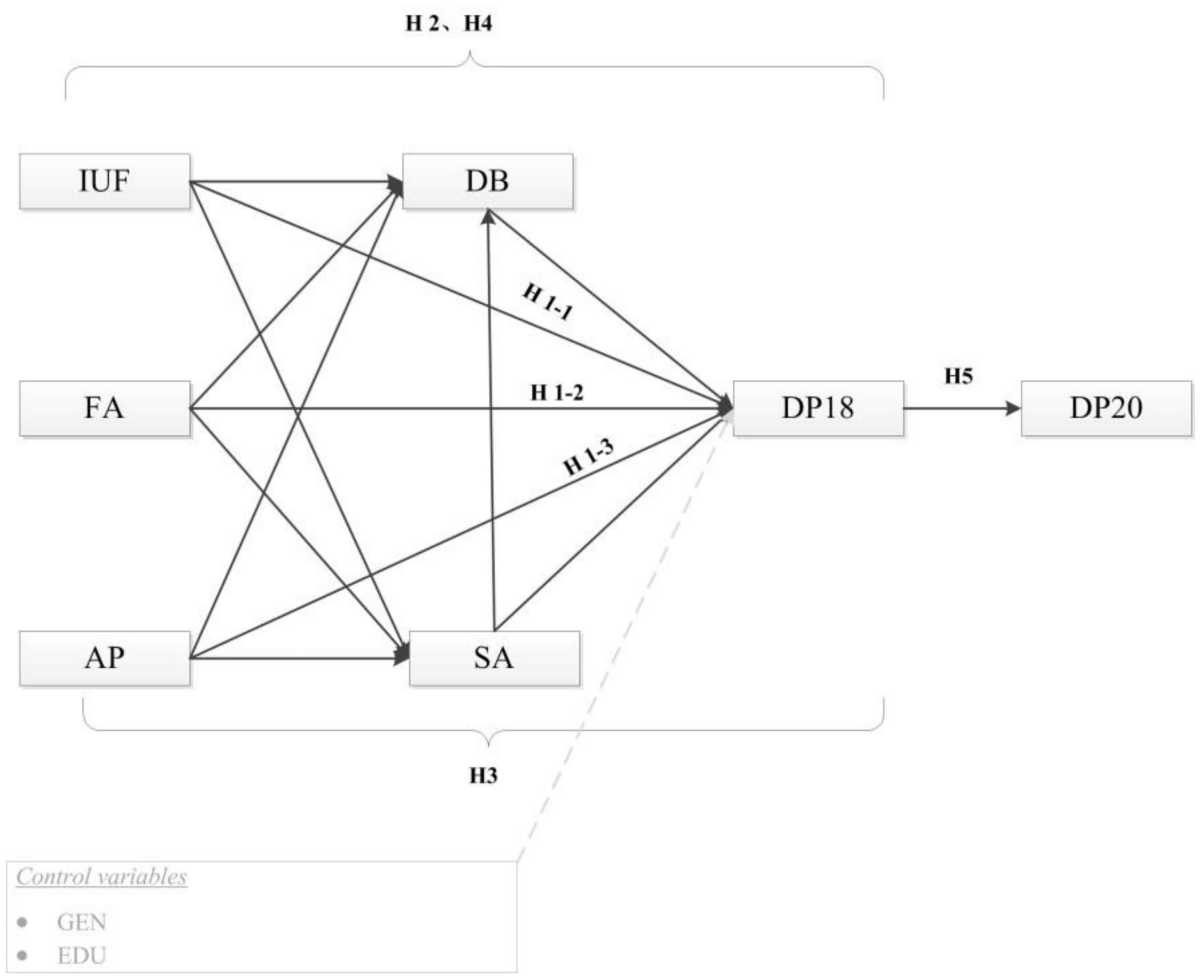
Hypothesized model of the research framework. IUF, Internet use Frequency; FA, Family Atmosphere; AP, Academic Performance; SA, Self-adjustment; DB, Deviant Behavior; DP18, Depression in 2017–2018; DP20, Depression in 2019–2020.

What is the relationship between the frequency of Internet use, family atmosphere, academic performance, and depression among adolescents? Do self-adjustment and campus deviant behavior have a mediating effect on adolescent depression? Can adolescents’ depression in 2018 affect their depression in ServanMori et al. (2020)? Based on the existing theoretical basis and literature, the research hypothesis is shown in Figure 1: (H1-1) Internet use frequency is positively correlated with adolescent depression; (H1-2) Family atmosphere is negatively correlated with adolescent depression; (H1-3) Academic performance is positively correlated with adolescent depression; (H2) Campus deviant behavior has a potential mediating role in the relationship between frequency of Internet use, family atmosphere, academic performance, and adolescent depression; (H3) Self-adjustment plays a potential mediating role in the relationship between Frequency of Internet use, family atmosphere, academic performance, and adolescent depression; (H4) campus deviant behavior and self-adjustment have chain mediating effects in the relationship between Internet use frequency, family atmosphere, academic performance, and adolescent depression; and (H5) There are persistent effects in adolescent depression.

## Materials and methods

### Participants

**Figure 2:**
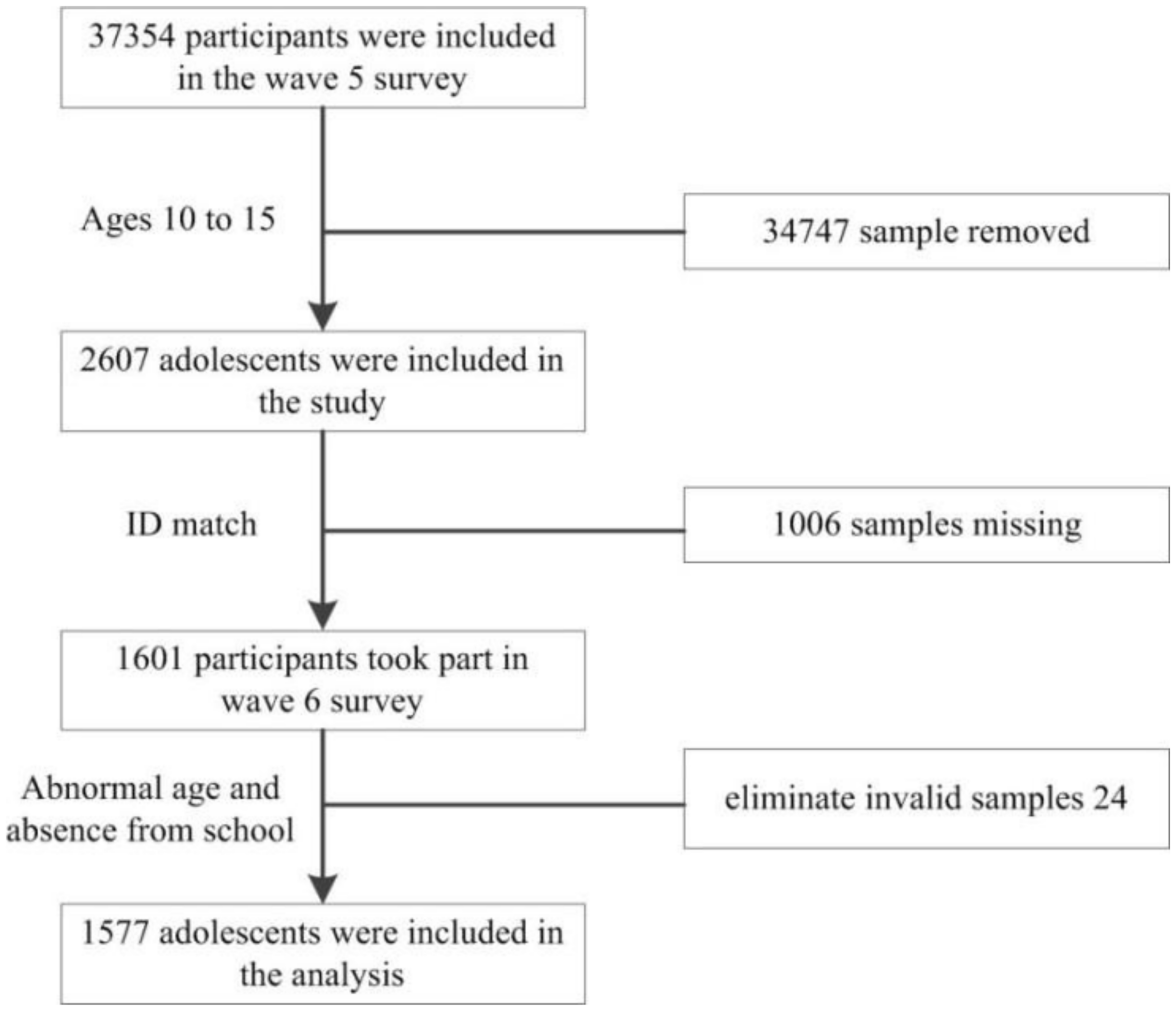
Flowchart of the inclusion and exclusion of participants.

The data are from China Family Panel Studies (CFPS) in The 5th Wave (2017–2018) and the sixth wave (2019–2020). CFPS is a nationwide, large-scale, and multi-disciplinary social follow-up survey project, which mainly covers the subjects of Chinese residents’ economic activities, educational achievements, family relationships, family dynamics, health, etc. And that baseline survey was officially carried out in 25 provinces/municipalities/autonomous regions, Finally, 14,960 households and 42,590 individuals were interviewed, which is the permanent tracking object of the CFPS survey and visited every 2 years. The sample selection of this study is shown in Figure 2. First of all, a total of 37,354 people participated in the 5th Wave survey. According to the characteristics of the research subjects, 34,747 people were selected, and then 1,006 people were selected according to the ID of The 5th Wave and the sixth wave of survey subjects. In addition, after eliminating 7 people with abnormal age and 17 people who did not go to school in two waves, a final sample of 1,577 people was obtained.

### Measures

#### Internet use frequency

The frequency of Internet usage is mainly measured by the following questions. (1) the frequency of using the Internet to socialize; (2) the frequency of using the Internet for entertainment; and (3) the Frequency of Internet business activities. Answers are divided into 7 levels (1 = never, 2 = once every few months; 3 = once a month; 4 = 2–3 times a month; 5 = 1–2 times a week; 6 = 3–4 times a week; 7 = almost every day). The total score for frequency of Internet use is between 3 and 21 points. The higher the score, the more frequently the Internet is used.

#### Family atmosphere

The family atmosphere of this study is mainly divided into two parts: parent–child relationship and parent–child interaction. In order to measure the parent–child relationship, this study uses the following questions: “the degree of trust in parents,” and the answers include five levels (1 = very distrust, 2 = distrust; 3 = average; 4 = Trust; 5 = very trusting). To measure parent–child interaction, participants were asked the following questions: “How many times have you talked to your parents in the past month” and “How many times have you had dinner with your family in the past week,” the answers include five levels (1 = never; 2 = 1 ~ 2 times, 3 = 3–4 times, 4 = 5–6 times, 5 ≥ 7 times).

#### Academic performance

The academic performance in the study mainly includes objective academic performance and subjective self-evaluation. The objective academic performance includes the following two questions: “class ranking” and “grade ranking.” The question options are divided into five grades (1 = the last 24%; 2 = 51–75%, 3 = 26%–50%, 4 = 11%–25%, and 5 = the top 10%). Subjective self-evaluation includes: “academic satisfaction” (1 = very dissatisfied, 2 = dissatisfied, 3 = average, 4 = satisfied, and 5 = very satisfied) and “How good do you think you are” (1 = very not good, 2 = not excellent, 3 = average, 4 = excellent, and 5 = very excellent) two questions.

#### Self-adjustment

Since 2012, CFPS has investigated the self-adjustment ability of adolescents aged 10 to 15 in their personal database. This scale mainly consists of 12 items, which are used to evaluate the self-adjustment state of daily behavior. Mainly include: I am always well prepared, I pay attention to details, I like to be organized, I will do things according to my own schedule, I am very careful in my study, I always put things at random, I always mess things up, I always forget to restore them, I do things carefully and thoroughly, I do my homework first and then play, and my homework assignment. I’ll start right after “and” I’ll clean up when things get messy “twelve questions.” Questions 6 (I always put things at random), 7 (I always mess things up), and 8 (I always forget to restore things) are reverse questions, and their answers are scored in reverse before analysis. Each item is rated from 1 to 5, where 1 = “strongly disagree,” 2 = “disagree,” 3 = “neither agree nor disagree” 4 = “agree,” and 5 = “quite agree.” The higher the score, the stronger the self-adjustment ability. The Cronbach alpha coefficient of the self-adjustment scale in this study is 0.871.

#### Adolescents’ campus deviant behavior

In the CFPS2018 Personal Questionnaire, CFPS collected information about adolescent respondents aged 10 to 15 for the first time, which contains 14 questions, including 8 internalized questions and six externalized questions. Among them, the internalization of adolescent campus deviant behavior is collinear with depression, so this study mainly uses six externalization problems to measure adolescents’ campus deviant behavior, including quarreling, attention, distraction, homework completion, talkativeness, and fighting. Each entry is rated from 1 to 5, where 1 = “completely non-conforming” and 2 = “non-conforming,” 3 = “average,” 4 = “relatively consistent,” and 5 = “completely consistent.” The higher the score, the greater the probability of adolescents’ campus deviant behavior. The Cronbach alpha coefficient of campus deviant behavior in this study is 0.742.

#### Depressive symptoms

CES-D scale is one of the most widely used scales for measuring depressive symptoms in the world. At present, CES-D is widely used in large-scale international surveys. CES-D scale is not only suitable for adults, but also for adolescents and the elderly. Its measurement contents include depression symptoms such as depression, feeling of worthlessness, despair, loss of appetite, and poor attention (Kohout et al., 1993; Wang et al., 2019; Liu et al., 2020). The original version of the CES-D scale includes 20 questions, but there are also shorter versions, one of which is 11 questions and 8 questions edited by HRS according to the original version (Michelle and Marc, 1999). This CES-D scale consists of 8 items, which evaluate the depressive symptoms of adolescents in the past week, including 6 positive items: “I feel depressed,” “I feel it’s hard to do anything,” “I feel bad sleep,” “I feel lonely,” “I feel sad,” and “I feel life cannot move on.” There are two negative entries: I feel happy and I live happily. The answer includes four grades: 1 = almost nothing (less than a day); 2 = Sometimes (1–2 days); 3 = Frequently (3–4 days); and 4 = Most of the time (5–7 days), reverse questions are negatively scored, and the total score ranges from 8 to 32. The higher the score, the more serious the depressive symptoms are. Cronbach alphas coefficients of the 2018 Depression Scale and the 2020 Depression Scale in this study are 0.752 and 0.812, respectively.

### Data analysis

Python3.9, SPSS22, and LISREL8.80 software were used for statistical analysis. Python3.9 was used to combine Wave5 and Wave6 data based on personal ID. SPSS was used to analyze the correlation between variables. Cronbach alpha coefficient was used to evaluate the internal consistency of the scale, and LISREL was used to construct chain structure equation. Frequency was used in counting data, mean and standard behavior was used in measuring data, and the structural equation model is used to test the intermediary effect. Mediation variables are self-adjustment and campus deviant behavior; Independent variables are Internet use frequency, family atmosphere, and academic performance; Dependent variables are depressive symptoms in 2018 and depressive symptoms in 2020 (Figure 1). When the values of comparison fitting index (CFI), non-normed fitting index (NNFI), incremental fitting index (IFI), and modified goodness of fit index (AGFI) are higher than 0.90, it indicates that the fitting results of the data are good (Bentler, 1990; Hu and Bentler, 1999), The approximate root means error (RMSEA) value < 0.05 means “close fit” (Steiger, 1990; Browne and Cudeck, 1992). The critical value (CN) of Hoelter greater than 200 indicates that the model has a good fitting degree (Bollen, 1986).

## Results

### Descriptive data

Table 1 shows the main demographic characteristics of the respondents. Among the 1,577 participants, there is little difference in the ratio of males to females. Most of the respondents attend primary schools. Among the frequency of Internet use, 18.71% use the Internet to socialize almost every day and 19.40% use the Internet for entertainment almost every day. In the family atmosphere, the average score of trust in parents is 4.76 (SD = 0.62), 55.51% of adolescents have never talked to their parents for nearly a month, while 1.98% of participants have never had dinner with their families for nearly a week; In terms of academic performance, 26.12% and 20.98% of adolescents ranked in the top 10% of classes and grades, respectively. 11.99% of the respondents were very satisfied with their self-study, and 5.08% of adolescents thought they were excellent. The average score for self-adjustment is 42.40 (SD = 6.79), the average score for campus deviant behavior is 12.59 (SD = 4.00), the average score of depressive symptoms in 2018 was 11.88 (SD = 3.04), and the average score for depressive symptoms in 2020 was 7.64 (SD = 2.20).

**Table 1:** Descriptive statistics variables of the sample (*n* = 1,577).

Variable	*n* ^#^	%	Mean	SD
**Control variable**				
**Sex**				
Male	803	50.92		
Female	774	49.08		
**Education**				
Primary school	956	60.62		
Junior school	592	37.54		
Technical secondary school/high school/higher vocational school or above	29	1.84		
**Independent variables**				
Frequency of Internet use [3–21]			8.68	5.44
The frequency of using the Internet to socialize				
Never	780	49.46		
Once every few months	25	1.59		
Once a month	17	1.08		
2–3 times a month	57	3.61		
Once or twice a week	192	12.18		
3–4 times a week	211	13.38		
Almost every day	295	18.71		
**Frequency of using the Internet for entertainment**				
Never	673	42.68		
Once every few months	19	1.20		
Once a month.	20	1.27		
2–3 times a month	65	4.12		
Once or twice a week	223	14.14		
3–4 times a week	271	17.18		
Almost every day	306	19.40		
**Frequency of Internet business activities**				
Never	1,290	81.80		
Once every few months	61	3.87		
Once a month.	58	3.68		
2–3 times a month	82	5.20		
Once or twice a week	56	3.55		
3–4 times a week	24	1.52		
Almost every day	6	0.38		
**Family atmosphere**				
Trust in parents [1–5]			4.76	0.62
The number of times parents talk				
Never	872	55.51		
1~2 times	299	19.03		
3–4 times	198	12.60		
5–6 times	103	6.56		
≥7 times	99	6.30		
**Number of dinners with family members**				
Never	31	1.98		
1~2 times	255	16.26		
3–4 times	97	6.19		
5–6 times	49	3.13		
≥7 times	1,136	72.45		
**Academic performance**				
**Class rank**				
The last 24%	101	7.83		
51%–75%	148	11.47		
26%–50%	353	27.36		
11%–25%	351	27.21		
Top 10%	337	26.12		
**Grade ranking**				
The last 24%	82	7.61		
51%–75%	187	17.36		
26%–50%	320	29.71		
11%–25%	262	24.33		
Top 10%	226	20.98		
**Academic satisfaction**				
Very dissatisfied	68	4.31		
Dissatisfied	122	7.74		
common	771	48.92		
be satisfied	426	27.03		
Very satisfied	189	11.99		
**Think how good you are**				
Not very good	62	3.93		
Not good	196	12.44		
common	855	54.25		
excellent	383	24.30		
Very good	80	5.08		
**Mediating variables**				
Self-adjustment [12–60]			42.40	6.79
Campus deviant behavior [6–30]			12.59	4.00
**Dependent variable**				
Depression 2018 [8–32]			11.88	3.04
Depression 2020 [8–32]			7.64	2.20

# The total number < *n* = 1,577 due to missing. [], The range of a single item.

### Mediation analyses

**Figure 3:**
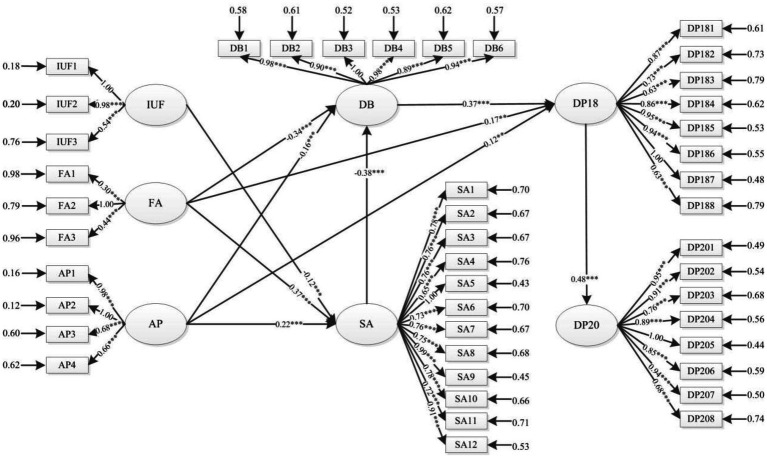
A Chain-mediated model of Self-adjustment and Campus Deviant Behavior on Adolescent Depression. IUF, Internet use Frequency; FA, Family Atmosphere; AP, Academic Performance; SA, Self-adjustment; DB, Deviant Behavior; DP18, Depression in 2017–2018; DP20, Depression in 2019–2020; 2019–2020; **p < 0.01, ***p < 0.001.

According to the structural equation model, the insignificant path is removed from the initial model by t value (t < 1.96) to get the final model. Compared with the initial model, the fitting result is improved to some extent, RMSEA = 0.046, NNFI = 0.90, CFI = 0.90, IFI = 0.90, AGFI = 0.94, and CN = 0.94. The family atmosphere has a significant negative impact on campus deviant behavior and 18-year depression, and a significant positive impact on self-adjustment. Academic performance has a significant negative effect on adolescent campus deviant behavior and a significant positive effect on self-adjustment and depression. Self-adjustment is directly and negatively related to adolescent campus deviant behavior; campus deviant behavior was positively correlated with depressive symptoms at 18 years. At the same time, 18-year depression symptoms of adolescents are directly and positively correlated with 20-year depression symptoms (Figure 3; Table 2).

**Table 2:** Measures of goodness-off-fit for depression model of the adolescents.

Model	Chi-Square	df	RMSEA	NNFI	CFI	IFI	AGFI	CN
Initial model	2508.88	1,044	0.046	0.90	0.90	0.90	0.94	309.89
Delete IUF → DB	2510.43	1,045	0.046	0.90	0.90	0.90	0.94	309.98
Delete GEN → DP18	2511.86	1,046	0.046	0.90	0.90	0.90	0.94	310.09
Delete IUF → DP18	2512.20	1,047	0.046	0.90	0.90	0.90	0.94	310.33
Delete SA → DP18^#^	2512.53	1,048	0.046	0.90	0.90	0.90	0.94	310.57

IUF, Internet use Frequency; DB, Deviant Behavior; GEN, gender; SA, Self-adjustment; DP18, Depression in 2017–2018.

# The goodness-of-fit of the Final model.

First of all, Table 3 lists the paths of various factors affecting adolescent depressive symptoms. The study found that Internet use frequency has a positive effect on adolescent depression symptoms, with a total effect of 0.02 (*p* < 0.001), but no direct effect. The family atmosphere has a direct influence on adolescent depression symptoms (*β* = −1.07, *p* < 0.001), and the total effect is-1.25 (*p* < 0.001); at the same time, we found that academic performance had a significant direct impact on adolescent depression (β = 0.12, *p* < 0.005). Due to the influence of intermediary factors, the total effect decreased to 0.03 (*p* < 0.001). Therefore, hypothesis 1(H1) is partially supported.

**Table 3:** Direct and indirect effects of depression in adolescents.

Variables	DP in 2018			DP in 2020		
	Direct effect	Indirect effect	Total effect	Direct effect	Indirect effect	Total effect
**Independent variables**						
Internet use frequency	−	0.02	0.02	−	0.01	0.01
Family atmosphere	−1.07	−0.18	−1.25	−	−0.60	−0.60
Academic performance	0.12	−0.09	0.03	−	0.02	0.02
**Mediation variables**						
Self-adjustment	−	−0.14	−0.14	−	−0.07	−0.07
Campus deviant behavior	0.37	−	0.37	−	0.18	0.18

Secondly, the results show that campus deviant behavior of adolescents has a direct impact on depression symptoms in DP18 (*β* = 0.37, *p* < 0.001) and a negative impact on depression symptoms in DP20, with a total effect of-0.07 (*p* < 0.001). Self-adjustment has no direct effect on adolescents’ depressive symptoms in DP18, but the total effect is-0.14 (*p* < 0.001), and it has a significant positive effect on adolescents’ depressive symptoms in DP20, with the total effect being 0.18 (*p* < 0.001). And self-adjustment has a significant negative influence on campus deviant behavior of adolescents (β = −0.38, *p* < 0.001). In addition, Internet use frequency, family atmosphere, and academic performance all have indirect effects on adolescents’ 20-year depression symptoms, and the total effects are −0.60, 0.01, and 0.02, respectively (*p* < 0.001). Therefore, Hypotheses 2, 3, and 4 (H2, H3, and H4) are supported to some extent.

Finally, the study shows that the depressive symptoms of adolescents are persistent over time, specifically, 18-year depressive symptoms have a significant positive effect on 20-year depressive symptoms, with a total effect of 0.48 (*p* < 0.001), which indicates that adolescents suffering from depressive symptoms in 18 years are more likely to suffer from depressive symptoms in 20 years. Therefore, hypothesis 5 (H5) is supported.

## Discussion

The chain mediation model was used to test the influence of Internet use frequency, family atmosphere, and academic performance on adolescent depression. The results showed that Internet use frequency had no direct influence on adolescent depression, the family atmosphere had a negative correlation with depressive symptoms, and the academic performance had a positive correlation with depressive symptoms. Intermediary effect discovery, Internet use frequency, family atmosphere, academic performance, and depressive symptoms can be influenced by independent mediation of campus deviant behavior and chain mediation of self-adjustment and campus deviant behavior. At the same time, this study also found that the depressive symptoms of adolescents are persistent over time.

### Internet use frequency can indirectly affect adolescent depressive symptoms

It is found that the total effect of Internet use frequency on adolescent depression is 0.02 (*p* < 0.001), which indicates that there is a certain correlation between them. More frequent internet usage, the higher the adolescent depression. Some studies show that people with high depression levels have high anxiety and low self-confidence in social interaction, and are often reluctant to participate in face-to-face social activities. Spending too much time on the Internet will inevitably reduce their social participation, thus making it impossible to obtain enough social support and emotional comfort (Cao et al., 2020). At the same time, low social interaction will also reduce the social belonging of adolescents, thus increasing the risk of depression. In addition, some related studies show that when the individual’s psychological demands cannot be met in face-to-face communication, it will alleviate psychological deficiency through network channels (Pontes, 2017), but the results of this study confirm that virtual network cannot make up for the psychological deficiency of adolescents, but will aggravate the level of depression. Therefore, it is suggested that the school joint family should further limit the frequency of adolescents using the Internet, guide adolescents to have a correct view of the Internet, appropriately strengthen social activities, and alleviate their emotional problems. In addition, the study found that the frequency of Internet use cannot directly affect the campus deviant behaviors of adolescents, but it can indirectly affect deviant behaviors on campus by reducing self-adjustment. This suggests that adolescents with better self-adjustment will actively control their own use of the Internet, thereby reducing deviant behavior.

### Family atmosphere can effectively relieve adolescent depressive symptoms

It is proved that family atmosphere has a reverse effect on adolescents’ depression, with a total effect of −1.25 (*p* < 0.001) and a direct effect of −1.07 (p < 0.001). Therefore, it can be seen that the adolescents with a better family atmosphere have a lower risk of depression, which is consistent with the existing research results (Ribeiro et al., 2016) As a special group, adolescents’ personality characteristics are not sound enough, their ability to cope with setbacks is lacking, their self-defense mechanism is not sound enough, their sense of social responsibility is relatively lacking, and they are prone to negative emotions leading to depression (Gunnell et al., 2018). And adolescence is the key period of life development and values establishment. How to guide adolescents to form the correct outlook on life and values, The formation of sound psychological characteristics and the avoidance of depression cannot be separated from a harmonious family atmosphere (Kenan and Aysel, 2012), and at the same time, the family function has a strong constraint on the individual’s psychological state and behavior to a certain extent (Edel and Brendan, 2013). Therefore, we should pay attention to the family environment, create a harmonious family atmosphere and improve the socialization function of the family. Provide a solid support for the growth of adolescents.

### Academic performance is an important factor affecting adolescent depression

Through model verification, the study found that academic performance has a positive effect on adolescent depression, with a direct effect of 0.12 (*p* < 0.001) and a total effect of 0.03 (*p* < 0.001). This shows that the better academic performance, the greater the risk of depression, and the intermediary factors can effectively alleviate the influence of academic performance on adolescent depression. The better academic performance, the higher the expectation, When academic achievement is contrary to effort and ideal expectation, self-confidence is more easily impacted, which leads to pessimism and negative emotions (Khan et al., 2022). Secondly, adolescents’ self-adjustment ability is relatively lacking, and their psychological resilience is relatively weak. When the learning pressure is too high, and they lose their self-worth, if they are not given psychological counseling in time, they are more likely to go to extremes (Zhu et al., 2021). In addition, Most Chinese parents have the mentality of “looking forward to their children’s success.” When their children’s academic performance falls short of their expectations, they tend to show irritability, anxiety, and other emotions, which in turn leads to parent–child conflicts and increases the risk of depression among adolescents (Warikoo et al., 2020). Therefore, it is necessary to strengthen the psychological construction of adolescents, at the same time reduce their learning pressure and establish correct values for adolescents.

### Chain mediating effect of self-adjustment and campus deviant behavior

The results show that self-adjustment and campus deviant behavior have a significant chain mediating effect, Among them, self-adjustment has a significant negative impact on campus deviant behavior, and campus deviant behavior has a significant positive impact on adolescent depressive symptoms. First, the control and restraint of adolescent s’ online behavior and online frequency can reduce the frequency of Internet use, thereby reducing campus deviant behavior; The control of self-emotion can ease the contradiction between parents and children; The regulation of cognitive activities can also relieve learning pressure and thus reduce campus deviant behavior of adolescents. Adolescents with high self-adjustment can actively adjust their emotions, behaviors, and cognition according to their goals, which can also effectively reduce the risk of depression (Finning et al., 2017). Secondly, To reduce adolescents’ campus deviant behavior, to some extent, it is necessary to enhance their social adaptability and anti-frustration ability, which can effectively relieve depression (Chen and Lien, 2018). Therefore, it is necessary to strengthen the training of adolescents’ self-adjustment ability, thereby increasing the effectiveness of Internet use and reducing aggressive behavior. At the same time, we should focus on adolescents’ anti-frustration ability and psychological resilience. Starting from emotional self-adjustment, promoting adolescent’s personality health. However, self-adjustment has no direct effect on depression, and the theory of self-adjustment resource depletion also indicates that the individual’s self-adjustment ability is limited and weakens with use (Baumeister et al., 2007), and appropriate self-adjustment is the protection for relieving depression factors, and excessive self-adjustment can easily lead to depression.

### The persistence of depressive symptoms in adolescents

This study also found that the depressive symptoms of adolescents persist in time, which is consistent with the previous research results (Lee et al., 2020; Li et al., 2021). That is to say, Depression of adolescents in 2017–2018 will affect depression in 2019–2020 to a certain extent. At the same time, depression is also affected by other factors, such as personality, emergencies, stress, etc., and effective self-adjustment and a harmonious family environment are protective factors for relieving depression. Therefore, from the perspective of prevention, early detection and early guidance should be made. At the same time, we should choose diversified educational paths, Create an elastic psychological state. Actively respond to the combination of home and school, and take the premise of respecting adolescents’ physical and mental development, so that they can establish correct values, outlook on life, and world outlook.

## Limitations

Some limitations to this study warrant consideration. First, Internet use frequency, family atmosphere, academic performance, self-adjustment, campus deviant behavior, and depression are cross-sectional in the study and can be further validated using longitudinal data in the future. Second, since the information was gathered from the participants in the study, self-report/recall bias may have existed. However, it is not easy to achieve continued participation among cohorts of adolescents in a cohort study, and the sample size should not be ignored. As a result, our findings with acceptable goodness-of-fit indices deserve paying more attention. In addition, Internet use frequency, family atmosphere, and academic performance have not been measured by relevant scales in the China Family Panel Studies database. This study only defines the concept according to relevant articles, and selects indicators according to the content of the database. The possible indicators are subjective, so more accurate and objective measurement is needed in later research.

## Conclusion

The depressive symptoms of adolescents should be paid constant attention, and the depressive symptoms are persistent on the time baseline. The chain mediating effect of self-adjustment and campus deviant behavior on depression among adolescents, Frequency of Internet use, family atmosphere, and academic performance are important factors affecting depressive symptoms.

## Data availability statement

The original contributions presented in the study are included in the article/supplementary material, further inquiries can be directed to the corresponding author.

## Ethics statement

This study was approved by the Ethical Review Committee of Peking University Biomedical (IRB00001052-14010), and all participants signed the informed consent. Written informed consent to participate in this study was provided by the participants' legal guardian/next of kin.

## Author contributions

M-mJ and YK designed the study, analyzed results, and drafted and revised the manuscript. D-wW and Z-yW drafted and revised the manuscript. KG and P-pG analyzed results and revised the manuscript. YK acquisition of funding. All authors contributed to the article and approved the submitted version.

## Funding

This study was supported by the National Natural Science Foundation of China (no. 72274023), Ministry of Education Humanities and Social Sciences Foundation of China (no. 22YJA890037), Social Science Planning Fund of Shandong Province, China (no. 22CGLJ01), and Natural Science Foundation of Shandong Province, China (no. ZR2022MG037).

## Conflict of interest

The authors declare that the research was conducted in the absence of any commercial or financial relationships that could be construed as a potential conflict of interest.

## Publisher’s note

All claims expressed in this article are solely those of the authors and do not necessarily represent those of their affiliated organizations, or those of the publisher, the editors and the reviewers. Any product that may be evaluated in this article, or claim that may be made by its manufacturer, is not guaranteed or endorsed by the publisher.

## Abbreviations

IUF: Internet use frequency
FA: Family atmosphere
AP: Academic performance
SA: Self-adjustment
DB: Deviant behavior
DP18: Depression in 2017–2018
DP20: Depression in 2019–2020
M: Mean
SD: Standard behavior
DE: Direct effect
IE: Indirect effect
TE: Total effect
SEM: Structural equation modeling
